# Establishment and assessment of a nomogram for predicting blood transfusion risk in posterior lumbar spinal fusion

**DOI:** 10.1186/s13018-020-02053-2

**Published:** 2021-01-11

**Authors:** Haosheng Wang, Kai Wang, Bin Lv, Haotian Xu, Weibo Jiang, Jianwu Zhao, Mingyang Kang, Rongpeng Dong, Yang Qu

**Affiliations:** 1 Department of Orthopedics, The Second Hospital of Jilin University, 218 Ziqiang Street, Changchun, 130041 Jilin Province People’s Republic of China; 2 Department of Orthopedics, The Affiliated People’s Hospital of Jiangsu University, Zhenjiang, Jiangsu Province People’s Republic of China

**Keywords:** Blood transfusion, Blood loss, Lumbar fusion, Risk factors, Nomogram

## Abstract

**Background:**

The aim of this study was to determine the risk factors and develop a nomogram for blood transfusions after posterior lumbar spinal fusion (PSL).

**Methods:**

We conducted a retrospective, single-center study based on 885 patients receiving PSL, and data was obtained from May 2015 to September 2019. Univariable and multivariable logistics regression analysis were conducted to identify risk factors for blood transfusion, and a nomogram was constructed to individually evaluate the risk of blood transfusion. Discrimination, calibration, and clinical usefulness were validated by the receiver operating characteristics (ROC), C-index, calibration plot, and decision curve analysis, respectively. Bootstrapping validation was performed to assess the performance of the model.

**Results:**

Of 885 patients, 885 were enrolled in the final study population, and 289 received blood transfusion. Statistical analyses showed that low preoperative hemoglobin (Hb), longer time to surgery, operative time, levels of fusion > 1, longer surgery duration, and higher total intraoperative blood loss (IBL) were the risk factors for transfusion. The C-index was 0.898 (95% CI 0.847–0.949) in this dataset and 0.895 in bootstrapping validation, respectively. Calibration curve showed satisfied discrimination and calibration of the nomogram. Decision curve analysis (DCA) shown that the nomogram was clinical utility.

**Conclusions:**

In summary, we investigated the relationship between the blood transfusion requirement and predictors: levels of fusion, operative time, time to surgery, total intraoperative EBL, and preoperative Hb level. Our nomogram with a robust performance in the assessment of risk of transfusion can contribute to clinicians in making clinical decision. However, external validation is still needed in the further.

**Supplementary Information:**

The online version contains supplementary material available at 10.1186/s13018-020-02053-2.

## Background

Posterior spinal fusion (PSF) is a widely recognized surgical stabilization procedure in the treatment of various spinal diseases including deformity, tumor, degenerative disease, trauma, and infection [1, 2]. Intraoperative and/or post-operative blood loss was a major focus of spinal surgeons and anesthesiologist. In particular, complex spinal surgeries are performed frequently in the past few decades, and blood loss is still one of the most common complications in the procedure [3]. A recent investigation in the United States (US) has reported that the proportion of allogenic blood transfusion in patients who underwent PSL has been growing, which has doubled in the past 10 years [4]. Therefore, spinal fusion has been characterized as the top 10 surgical procedure in North America associated with blood transfusion—not surprisingly [5].

An investigation of spinal fusion has indicated that patients receiving blood transfusion had experienced adverse events and complications including longer hospital stay, higher incidence of surgical site infections, sepsis, febrile reactions, and pulmonary embolism [6, 7]. More importantly, blood transfusion would impose a huge burden on the individuals, families, and healthcare system worldwide, which remain an intractable health condition especially in areas lacking medical resources, especially in the developing regions and countries [8]. Hence, it is particularly important to identify that the risk factors in blood transfusion would help clinicians to evaluate the risk of individuals and make the best decision for each patient to minimize cost and reduce transfusion-related and other complications.

Regrettably, however, there is little evidence to explore the risk factors related to blood transfusion in patients receiving PSF. Aoude et al. indicated that age, dyspnea, ASA score, level of fusion, and high blood urea nitrogen (BUN) levels were the risk factors for blood transfusion [9]. Morcos et al. reported that ASA > 1, longer operating time, level of fusion > 1, sacrum inclusion, and open posterior approach were the significant risk factors for blood transfusion [10]. Hence, it is necessary to improve preoperative evaluation and blood management. Consequently, it is urgent for us to develop easy-to-use visualization tools for clinicians in clinical practice. Nomogram, a simple predictive tool, was widely used in various disciplines, which can help clinicians to estimate the probability of events [11, 12]. In this study, we aim to develop and validate a brief and reliable nomogram to evaluate the risk of blood transfusion in patients who underwent PSF and identify high-risk patients in clinical decision-making. Besides, we assess the accuracy of the nomogram via cross-validation and bootstrap of the data set.

## Methods

### Patients and data collection

**Fig. 1:**
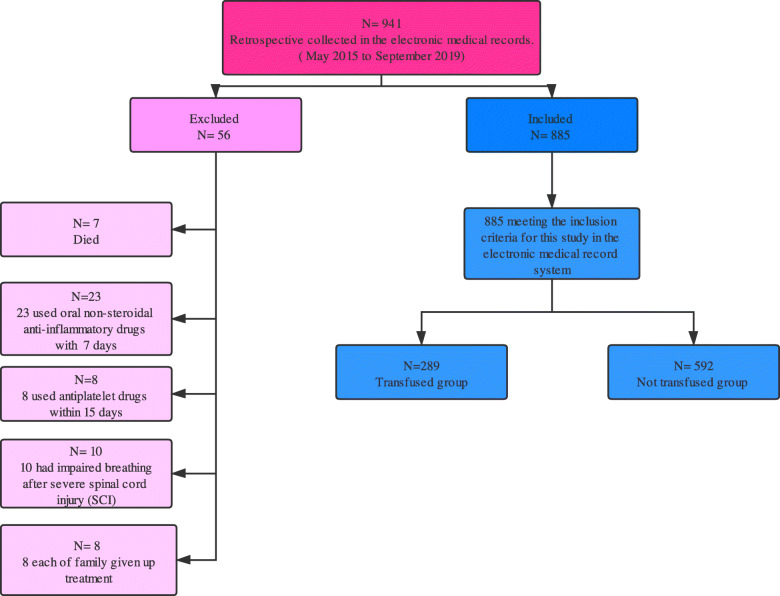
The flowchart of case inclusion and exclusion criteria

**Fig. 2:**
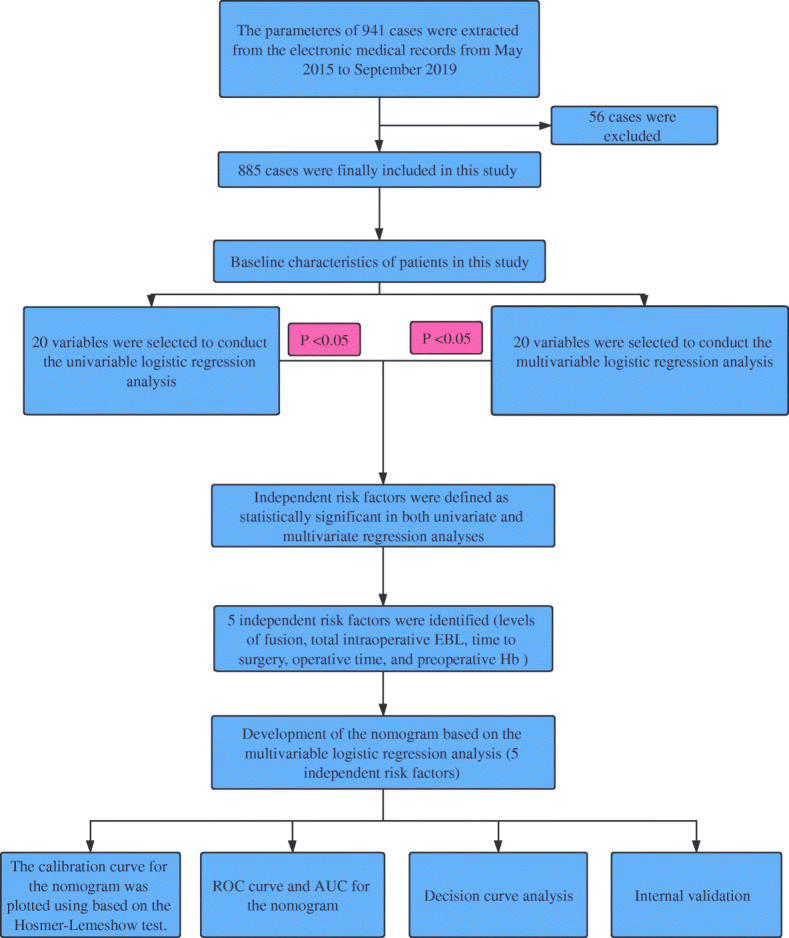
The flowchart of this study

This research was approved by the Ethics Committee of the Second Hospital of Jilin University (Changchun, People’s Republic of China). All patients provided informed written consent. The study inclusion and exclusion criteria are listed in Table 1. From May 2015 to September 2019, 941 patients, including 456 male patients and 485 female patients, were in compliance with the requirements. Of these, 56 were excluded: 7 died, 23 used oral non-steroidal anti-inflammatory drugs with 7 days, 8 used antiplatelet drugs within 15 days, 10 had impaired breathing after severe spinal cord injury (SCI), and 8 each of family given up treatment. Ultimately, 885 patients were included in this study (Fig. 1). All of the patients underwent a standard posterior spinal fusion. Although still controversial, careful preoperative preparation consists of the individualized assessment of the patient’s condition, grasping the appropriate timing of blood transfusion, and delicate intraoperative operation is still essential [13, 14]. Therefore, based on the experience in this centre, we conducted this study. The flowchart of the study was presented in Fig. 2.

**Table 1:** Inclusion criteria and exclusion criteria in this present study

**Number**	**Inclusion criteria**
1	(1) Lumbar disc herniation; (2) lumbar spinal stenosis; (3) lumbar spondylolisthesis; (4) lumbar disc herniation with spinal stenosis; (5) age greater than or equal to 40 years; (6) posterior fusion; (7) no coagulation abnormalities
	**Exclusion criteria**
2	(1) Revision surgery; (2) minimally invasive fusion surgery; (3) emergency surgery; (4) lumbar tumor; (5) lumbar tuberculosis; (6) Brucellosis; (7) lumbar scoliosis deformity; (8) lumbar fracture and dislocation; (9) cervical or thoracic surgery; (10) pre-deposit autologous blood transfusion; (11) recycled autologous blood transfusion; (12) preoperative blood transfusion

### Potential risk factors

Patient characteristics in this study including age, gender, body mass index (BMI), comorbid diseases (e.g., hypertension, congestive heart failure, bleeding disorder, and COPD), smoker, American Society of Anesthesiologists (ASA) class, levels of fusion, time to surgery, length of stay, total intraoperative estimated blood loss (EBL), operative time, previous transfusion, previous surgery, preoperative activated partial thromboplastin time (APTT), preoperative prothrombin time (PT), preoperative fibrinogen, preoperative platelet count, preoperative hematocrit, preoperative Hb, preoperative white blood cell count (WBC), and length of stay (LOS) were extracted from electronic medical records in our hospital.

### Statistical analysis

Data processing and statistical analysis were performed by the R software (Version 4.0.0; https://www.R-project.org). Among the clinical parameters, continuous variables were presented as median and interquartile range (IQR), while categorical variables were reported as integers and percentages. The distribution of categorical and continuous variables among groups was compared using Fisher’s exact test and Mann–Whitney *U* test, respectively. In order to further explore the potential relationship between the variables and blood transfusion, logistics regression analyses were carried out to control the confounding effects and identify the predictors of blood transfusion. Twenty variables were selected to conduct the univariable and multivariable logistics regression analysis. *P* value < 0.05 was considered significant in this study. Independent risk factors were defined as statistically significant in both univariate and multivariate regression analyses. Subsequently, we identified the independent risk factors related to blood transfusion in PSL. Apart from the statistical significance, we need to give priority to clinical significance of the independent risk factors. Ultimately, the selected variables were included to develop the newly built nomogram via multivariate regression analysis. The discrimination of the nomogram is defined as the ability to separate subject outcomes, which can be illustrated by the area under the receiver operating characteristic (ROC) curve (AUC). Meanwhile, the comparison of observed and predicted risk of blood transfusion is defined as calibration, which can be assessed by calibration plot, a graphical tool to demonstrate the nomogram calibration, and by calculating the Hosmer and Lemeshow goodness-of-fit test (HL test). A relatively corrected C-index (1000 bootstrap resamples) of the nomogram was also determined in this cohort. Decision curve analysis was used to evaluate the net benefit and usefulness of our model. In the process of DCA, the net benefit is calculated by subtracting the proportion of false-positive patients from the proportion of true positive patients in the population by a certain threshold that is set while taking into account the negative effects of not performing any non-essential intervention. The “rms”, “rmda”, and “nomogramEx” packages were used in the process.

## Results

### Patients’ characteristics

In this present study, we enrolled 885 patients with elective lumbar PSF, of whom 289 (32.7%) received blood transfusion. Table 2 demonstrates the baseline characteristics. Overall, 440 (49.718%) were males, and 445 (50.282%) were female. Among the clinical characteristics, gender (*p* = 0.312), comorbidities (*p* = 0.057), the proportion of smokers (*p* = 0.323), previous transfusion (*p* = 0.023), previous surgery (*p* = 0.211), preoperative APTT (*p* = 0.091), preoperative fibrinogen (*p* = 0.630), preoperative platelet count (*p* = 0.861), and preoperative WBC (*p* = 0.733), no statistical difference between the transfusion and no-transfusion group. Compared with transfusion cohort, the mean age was greater in the no-transfusion group. The preoperative Hb and hematocrit were statistically lower, while the BMI, operative time, time to surgery, total intraoperative EBL, level of fusion, ASA class, and length of stay were significantly higher in transfusion cohort than no-transfusion cohort. Other characteristics are demonstrated in Table 2.

**Table 2:** Patient demographics and preoperative characteristics

	Total	Not transfused	Transfused	*P* value
Number of patients	885	596	289	
Age (years)				0.038
< 60	339 (38.31%)	249 (41.78%)	90 (31.14%)	
≧ 60	546 (61.69%)	347 (58.22%)	199 (68.86%)	
Gender (%)				0.312
Male	440 (49.718%)	315 (52.852%)	125 (43.253%)	
Female	445 (50.282%)	281 (47.148%)	164 (56.747%)	
Comorbidities				0.057
None (%)	135 (15.254%)	93 (15.604%)	42 (14.533%)	
1–3 (%)	543 (61.356%)	352 (59.060%)	191 (66.090%)	
≧ 4 (%)	207 (23.390%)	151 (25.336%)	56 (19.377%)	
BMI (kg/m^2^)	28.8 [26.6, 31.0]	28.6 [26.4, 30.7]	29.6 [26.9, 32.1]	0.001
Smoker (%)				0.323
Yes	256 (28.927%)	79 (13.255%)	177 (61.246%)	
No	629 (71.073%)	517 (86.745%)	112 (38.754%)	
ASA class (%)				< 0.001
1	57 (6.441%)	49 (8.221%)	8 (2.768%)	
2	504 (56.949%)	351 (58.893%)	153 (52.941%)	
3	316 (35.706%)	192 (32.215%)	124 (42.907%)	
4	8 (0.904%)	4 (0.671%)	4 (1.384%)	
Levels of fusion (%)				< 0.001
1	344 (38.870%)	301 (50.503%)	43 (14.879%)	
2	184 (20.791%)	146 (24.497%)	38 (13.149%)	
≧ 3	357 (40.339%)	149 (25.000%)	208 (71.972%)	
Time to surgery (days)	5.4 [4.6, 6.5]	5.1 [4.3, 5.8]	6.5 [5.4, 7.7]	< 0.001
Total intraoperative EBL (ml)	357.9 [273.3, 512.0]	320.3 [238.3, 390.7]	857.3 [422.4, 956.1]	< 0.001
Operative time (min)	191.2 [154.3, 235.9]	181.0 [150.1, 210.5]	249.4 [176.7, 344.0]	< 0.001
Previous transfusion (%)				0.023
Yes	24 (2.712%)	11 (1.846%)	13 (4.498%)	
No	861 (97.288%)	585 (98.154%)	276 (95.502%)	
Previous surgery (%)				0.211
Yes	74 (8.362%)	45 (7.550%)	29 (10.035%)	
No	811 (91.638%)	551 (92.450%)	260 (89.965%)	
Preoperative APTT	31.4 [29.9, 33.0]	31.3 [29.9, 32.9]	31.6 [29.8, 33.6]	0.091
Preoperative PT	11.9 [11.1, 12.9]	12.1 [11.2, 13.1]	11.5 [10.8, 12.4]	< 0.001
Preoperative fibrinogen	3.2 [2.7, 3.7]	3.2 [2.7, 3.7]	3.2 [2.6, 3.7]	0.630
Preoperative platelet count	249.9 [211.3, 297.5]	248.3 [209.3, 298.9]	253.7 [214.8, 293.6]	0.861
Preoperative hematocrit	35.5 [32.9, 37.8]	36.4 [34.1, 38.6]	33.2 [31.0, 35.9]	< 0.001
Preoperative Hb	113.3 [106.2, 121.7]	115.5 [107.9, 126.2]	109.9 [104.2, 114.8]	< 0.001
Preoperative WBC	7.21 [5.6, 8.7]	7.2 [5.5, 8.6]	7.3 [5.7, 8.7]	0.733
LOS	9.12[5.6, 8.7]	7.13[4.90, 9.36]	11.77[6.93, 15.61]	< 0.001

*BMI* Body mass index, *ASA* American Stroke Association, *EBL* Estimated blood loss, *APTT* Activated partial thromboplastin time, *PT* Prothrombin time, *HB* Hemoglobin, *WBC* White blood cell count, *LOS* Length of stay

### Identification of the risk factors of blood transfusion

Based on the build cohort, 20 variables were selected to conduct the univariable and multivariable logistics regression analysis (Table 3). Combining clinical and statistical significance, 5 variables were finally identified as independent risk factors related to blood transfusion in PSL. Level of fusion (*β* 0.4421, OR 1.5560, 95% CI 1.2017–2.0187, *p* = 0.000815 **), time to surgery (*β* 0.2777, OR 1.3201, 95% CI 1.1014–1.5888, *p* = 0.002916 **), total intraoperative EBL (*β* 0.0067, OR 1.0067, 95% CI 1.0054–1.0082, *p* < 2e−16 ***), operative time (*β* 0.0046, OR 1.0047, 95% CI 1.0006–1.0089, *p* = 0.027468*), preoperative Hb (*β* − 0.0521, OR 0.9493, 95% CI 0.9309–0.9669, *p* = 6.92e−08 ***).The result of multivariable logistic regression analysis is illustrated in Table 4.

**Table 3:** Univariate and multivariate logistic regression analyses for predicting the blood transfusion risk in posterior lumbar spinal fusion

Variable	Univariable logistic regression analysis				Multivariable logistic regression analysis			
	Odds ratio	95% Confidence interval		***P*** Value	Odds ratio	95% Confidence interval		***P*** Value
		Lower	Upper			Lower	Upper	
**Age**	1.5866	1.1811	2.1422	0.00235 **	0.8805	0.5513	1.4092	0.59406
**Gender**	0.6799	0.5119	0.9016	0.00752 **	1.2191	0.7671	1.9530	0.40489
**Comorbidities**	0.8792	0.6993	1.1044	0.269	0.9960	0.6231	1.5799	0.98656
**BMI**	1.0834	1.0405	1.1288	0.000114 ***	1.0468	0.9763	1.1226	0.19802
**Smoker**	0.0967	0.0689	0.1346	< 2e−16 ***	0.4761	0.2262	0.9969	0.074
**ASA**	1.6408	1.2930	2.0899	5.20e−05 ***	1.2096	0.8331	1.7633	0.31869
**Levels of fusion**	3.3137	2.7367	4.0465	< 2e−16 ***	1.5165	1.1653	1.9773	0.00198 **
**Time to surgery**	2.3990	2.1007	2.7629	< 2e−16 ***	1.2902	1.0706	1.5617	0.00802 **
**Total intraoperative EBL**	1.0085	1.0074	1.0098	< 2e−16 ***	1.0065	1.0050	1.0081	5.15e−16 ***
**Operative time**	1.0156	1.0131	1.0184	< 2e−16 ***	1.0037	0.9997	1.0080	0.01582*
**Previous transfusion**	2.5049	1.1064	5.7728	0.0273 *	0.5880	0.0911	3.5178	0.58191
**Previous surgery**	1.3657	0.8296	2.2166	0.212	1.0246	0.4132	2.3364	0.95601
**Preoperative APTT**	1.0672	1.0054	1.1335	0.03318 *	0.9600	0.8688	1.0605	0.42184
**Preoperative PT**	0.7045	0.6280	0.7877	1.29e−09 ***	0.9916	0.8342	1.1773	0.92365
**Preoperative fibrinogen**	0.9480	0.7854	1.1438	0.5769	1.1889	0.8591	1.6510	0.29851
**Preoperative platelet count**	1.0003	0.9981	1.0025	0.7849	1.0021	0.9987	1.0056	0.22155
**Preoperative hematocrit**	0.7882	0.7516	0.8249	< 2e−16 ***	0.9804	0.9155	1.0494	0.56961
**Preoperative Hb**	0.9481	0.9353	0.9605	4.36e−15 ***	0.9491	0.9303	0.9671	1.26e−07 ***
**Preoperative WBC**	1.0044	0.9444	1.0681	0.8889	1.0533	0.9502	1.1671	0.32149
**LOS**	1.453	1.0287	1.8773	0.1342	1.324	1.0872	1.5608	0.081

*BMI* Body mass index, *ASA* American Stroke Association, *EBL* Estimated blood loss, *APTT* Activated partial thromboplastin time, *PT* Prothrombin time, *HB* Hemoglobin, *WBC* White blood cell count, *LOS* Length of stay

**Table 4:** Prediction factors for blood transfusion risk in posterior lumbar spinal fusion

Variable	β	Prediction model			
		Odds ratio	95% Confidence interval		***P*** Value
			Lower	Upper	
**Intercept**	− 1.3119	0.2693	0.0250	2.9201	0.279361
**Levels of fusion**	0.4421	1.5560	1.2017	2.0187	0.000815 **
**Total intraoperative EBL**	0.0067	1.0067	1.0054	1.0082	< 2e− 16 ***
**Time to surgery**	0.2777	1.3201	1.1014	1.5888	0.002916 **
**Operative time**	0.0046	1.0047	1.0006	1.0089	0.027468 *
**Preoperative Hb**	− 0.0521	0.9493	0.9309	0.9669	6.92e−08 ***

*EBL* Estimated blood loss, *Hb* Hemoglobin, *β* the regression coefficient

### Development of a prediction model

**Fig. 3:**
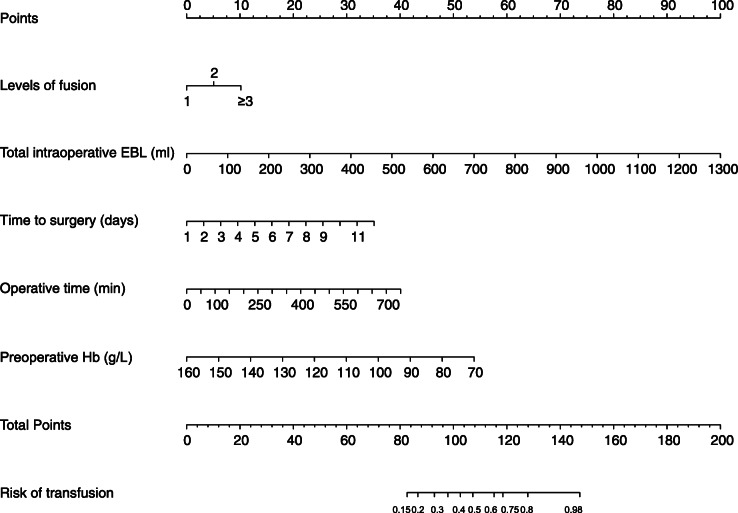
A nomogram to predict the risk of blood transfusions in PSL. EBL, estimated blood loss

Based on the result of multivariable logistic regression analysis, the following were related to blood transfusion: level of fusion, time to surgery, total intraoperative EBL, operative time, and preoperative Hb. These 5 factors were incorporated into the predictive model and develop a nomogram, a graphical toll, which can visualize the results of regression analysis (Fig. 3). The clinician can provide an individualized assessment of the risk of blood transfusion for patients undergoing PSL. For a specific patient, the total points were obtained by adding each score in the nomogram, and then, the corresponding transfusion probability of the patient can be obtained according to the total score. This will facilitate accurate preoperative risk assessment, better identification of the transfused population, and more efficient doctor-patient communication [15].

### Evaluation of the performance of the predictive model

**Fig. 4:**
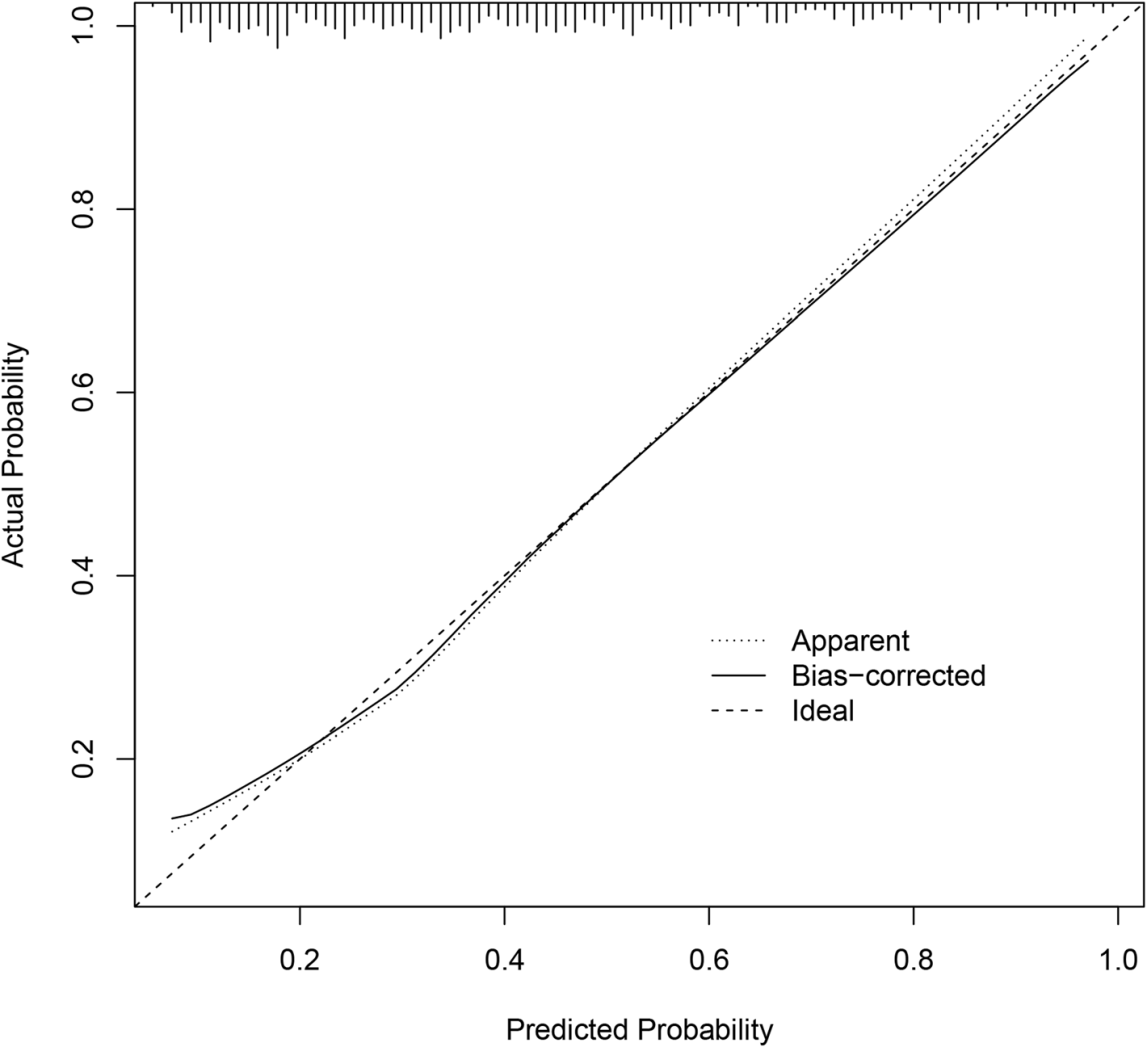
Calibration curve for nomogram prediction of risk of blood transfusions in posterior lumbar spinal fusion

**Fig. 5:**
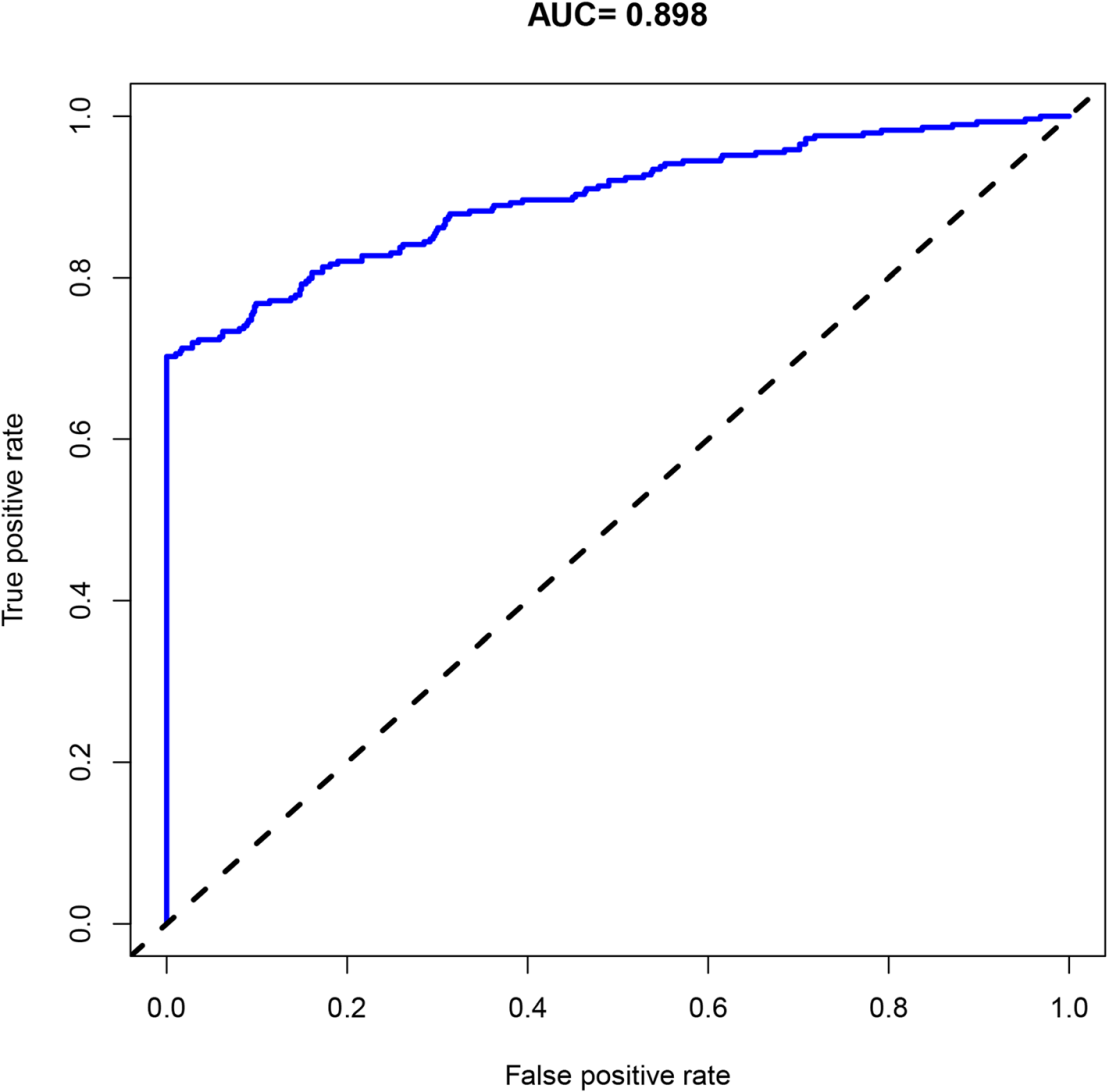
Receiver operating characteristic curve analysis—model validation

The calibration curve of the predictive model for evaluating the risk of transfusion in patients that underwent PSL showed satisfied agreement in this dataset (Fig. 4). Both discrimination and calibration of our model performed satisfactorily in a sizeable population of patients. Subsequently, the C-index of this model was 0.898 (95% CI 0.847–0.949) in this dataset and was an identified to be 0.895 via bootstrapping validation (Bootstrap = 1000). The ROC curve was generated, and AUC was identified as 0.898 in this dataset (Fig. 5). Overall, the nomogram demonstrated great performance in predicting the risk of blood transfusion.

### Clinical application

**Fig. 6:**
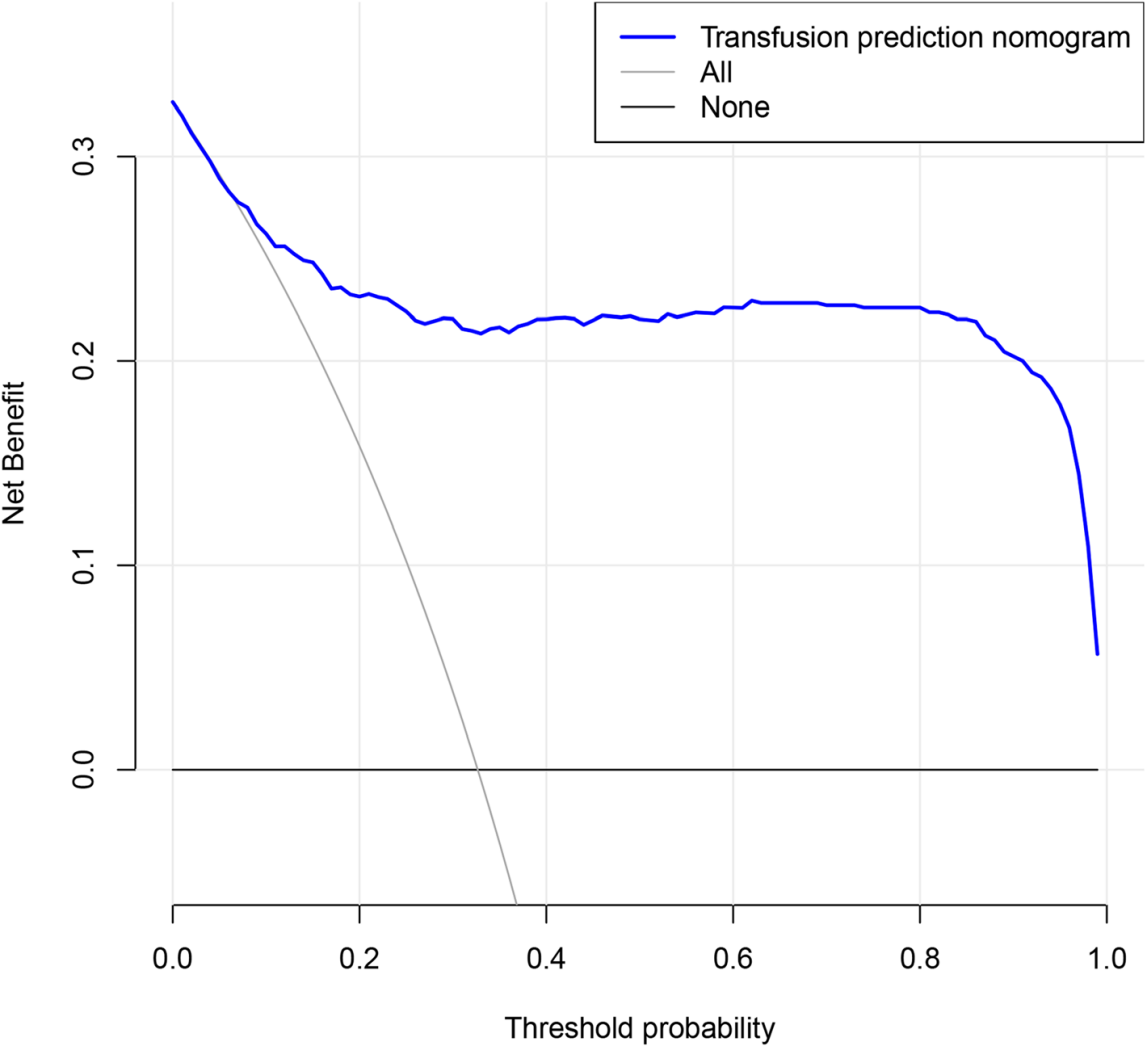
Decision curve analysis for nomogram prediction of risk of blood transfusions in posterior lumbar spinal fusion

To evaluate the clinical usefulness of the predictive model, a decision analysis (DCA) was performed in the data. The DCA is a novel method that assessed the clinical net benefit of the nomogram. The DCA is demonstrated in Fig. 6. For clinician and patients, if the threshold was set at 16% and above, the use of this model to predict the probability of patient transfusion is more beneficial than this scheme.

## Discussion

Posterior spinal fusion is the procedure of choice for patients with trauma, spinal infection, spinal deformities, and degenerative spinal diseases [16]. The total number of operations in PSL has been rising annually, especially in China [17, 18]. Significant blood loss is the most frequent surgical complication and receiving a great deal of attention in the spinal surgeon, as well as anesthesiologist [19]. There was no consensus among the definition of major blood loss; it is generally accepted that one volume of blood loss reaching the total blood loss (60 ml/kg adult) within 24 h was defined as major blood loss [20]. Now evolving techniques have helped the clinician to aid treatment decisions. Meanwhile, blood transfusion is by far the most effective way to treat the major blood loss in spinal surgeries [21]. However, blood shortage is an increasing problem within developing countries, especially in rural, underdeveloped areas of China [22]. Thus, early and accurate identification of the risk of blood transfusion is not only to save blood resources but also for better clinical outcome of patients. Although it has been recognized that PSL closely related to significant blood loss and transfusion, the related risk factors for transfusion were still unclear [10, 23]. In our cohort, 289 (32.7%) required blood transfusion. We identified independent risk factors associated with transfusion as follows: increased levels of fusion, prolonged operative time, longer time to surgery, total intraoperative EBL, and a low preoperative Hb level.

The preoperative Hb was 115.5 [107.9, 126.2] g/L in the not transfused group and 109.9 [104.2, 114.8] g/L in the transfused group (*p* < 0.001), respectively. Previous studies have reported that low preoperative Hb was the risk factor with a longer LOS, increased complications, higher costs, and increased mortality [24, 25]. Similarly, consistent with what we reported before, according to Perez et al., preoperative Hb was a critical predictor in the complex spine surgery [26]. These results corroborate the ideas of Morris et al., who suggested that patients with Hb < 120 g/L faced a significant risk of transfusion, with a risk that was approximately five times higher than the patients with Hb > 120 g/L. [27] It can thus be suggested that low preoperative Hb leads to poor immunity, and a poor tolerance had unfortunately resulted in low compensative ability to surgical and anesthetic trauma, as well as blood loss.

However, it was interesting that time to surgery was an independent risk factor of transfusion. Previous studies confirmed that the duration from admission to surgery was correlated with the incidence of complication, mortality, and clinical outcome [28]. Through the statistical analysis, we confirmed these conclusions; patients in this cohort who received transfusion had a length of stay which could be explained by poor preoperative general conditions. In not transfused group, the mean time to surgery was 5.1 [4.3, 5.8] days compared to 6.5 [5.4, 7.7] days in patients who received transfusion (*p* < 0.001). We cautiously assume that this may be because patients with delayed surgery have a series of reasons such as poor general condition, relatively complex surgery, or more complications, which might lead to challenging bleeding and need for transfusion.

In this present study, total intraoperative EBL was the strongest independent risk factor for transfusion. Although there is no consistent conclusion on the evaluation of blood loss and the indication of transfusion in spinal surgery, a considerable proportion of studies have reported that in cases requiring blood transfusion, the range of intraoperative blood loss is 650 to 2839 ml [20]. Moreover, even in the same diseases, we observed a significant positive relationship between blood loss and different procedures, including open surgery or minimally invasive surgery, and unilateral laminar fenestration decompression and fusion, laminar fenestration decompression and fusion, and total laminectomy and decompression. Kushioka et al. [29] conducted a randomized controlled study on blood loss during lumbar minimally invasive transforaminal lumbar interbody fusion (MIS-TLIF) and open transforaminal lumbar interbody fusion (TLIF), and reported that the total operative blood loss was 355 ml for MIS-TLIF and 538 ml for open TLIF. Zhang et al. [30] reported that the total intraoperative blood loss was 602 ml for lumbar MIS-TLIF and 42 ml for oblique lumbar interbody fusion (OLIF). Morcos et al. [10] reported after a retrospective analysis of transfusion risk factors in lumbar fusion surgery in Canada that perioperative blood transfusion was 18%, and intraoperative blood loss was 1018 ml (transfusion group) and 477 ml (non-transfusion group) in posterior lumbar fusion, respectively, and the difference in intraoperative blood loss between the two groups was statistically significant, but multivariate analysis showed that intraoperative blood loss was not a risk factor for perioperative blood transfusion. Therefore, intraoperative judgments of the operators were critical to the assessment of the risk of transfusion.

What attracted our attention is that operative time and numbers of levels of fusion were independent risk factors of transfusion, which was consistent with previous studies [10]. The risk of blood transfusion in 2 segments of posterior lumbar fusion was 1.5 times higher than that in 1 fusion, and the risk of blood transfusion in 3 or more segments of fusion was 3 times higher than that in 1 fusion. This confirms that there is a correlation between prolonged posterior spinal fusion surgery and increased operative blood loss [31]. Long fusion requires extensive exposure of the spine for pedicle screw placement and intraspinal decompression, which means that a large number of muscles and soft tissues behind the spine need to be dissected from bone tissue, and the more exposed the muscles, soft tissues, and bone surfaces, the more increased is the blood loss during the operation period. Morcos et al. [10] found that the increase of operative segment would prolong the operative time and then increase the risk of blood transfusion. Therefore, in the face of complex, more difficult, or more fused segments, we believe that good communication within the surgical team, between surgeons, operating room nurses, and anesthetists could reduce the operative time and incidence of transfusion.

Here, we develop a novel predictive nomogram for predicting the risk of transfusion in patients receiving PSL based on a single high-volume center for the first time in northeast China. Our nomogram can demonstrate all the key factors graphically and can individually evaluate the incidence of blood transfusions after lumbar PSF. This model can assist and contribute to clinical decision-making and identify the patients with a high risk of transfusion [32]. Additionally, it provides references for blood transfusion and saves blood resources and hospitalization costs [32, 33].

Several limitations of this study should not be ignored. First, this was a retrospective, single high-volume center study with possible bias, which limits its generality and weakens some statistical analyses. Second, external validation, especially in other regions and countries, in the future research is needed. Third, specific data could not be obtained from the medical records or were missing, including particular procedure, transfusion-related complications, intraoperative fluid infusion volume, and intraoperative urine volume.

## Conclusions

In summary, by using a single high-volume center for the first time in northeast China, we investigated the relationship between the blood transfusion requirement and predictors: levels of fusion, operative time, time to surgery, total intraoperative EBL, and preoperative Hb level. Our nomogram with a robust performance in the assessment of the risk of transfusion can contribute to clinicians making the clinical decision and determine whether individual based on our nomogram. However, external validation is still needed in the future.

## Supplementary Information

**Additional file 1.**

## Data Availability

All the data and materials can be found in the manuscript.

## Abbreviations

PSL: Posterior lumbar spinal fusion
BMI: Body mass index
ASA: American Stroke Association
EBL: Estimated blood loss
APTT: Activated partial thromboplastin time
PT: Prothrombin time
Hb: Hemoglobin
WBC: White blood cell count
TLIF: Transforaminal lumbar interbody fusion
MIS-TLIF: Minimally invasive transforaminal lumbar interbody fusion
OLIF: Oblique lumbar interbody fusion
