# Supplementary material for: Establishment and assessment of a nomogram for predicting blood transfusion risk in posterior lumbar spinal fusion
Source: J Orthop Surg Res. 2021 Jan 11;16:39. doi: 10.1186/s13018-020-02053-2 (PMC7798229; doi:10.1186/s13018-020-02053-2)
Supplement: Supplementary file 1 — Additional file 1. [file 13018_2020_2053_MOESM1_ESM.docx]

**Supplementary Materials**

This supplement details the issues mentioned in the Reviewer#3 comment 6.

Thank you for your careful work. Based on a retrospective cohort in Northeast China, we developed a nomogram to predict the blood transfusion risk in PSL via multivariate regression analysis. Based on your constructive comments, the methodological issue has been modified for better understanding as follows.

1. The workflow of this study was presented in Figure 1.

2. Introduction and implementation R code of statistical analysis

2.1 **Calibration curve**

**Function:** Model calibration (i.e., agreement between predicted and observed probabilities) was evaluated by visual inspection of the calibration plot and by computing the Hosmer and Lemeshow goodness-of-fit test (HL test). A significant HL test indicates evidence against good model fit.

**Input:** Dataset contains 885 cases parameters (Input 1.0.txt in the R code)

**Output: The calibration curve showed that** predicted probabilities were close to observed probabilities, and the coincidence was good (Figure 4).

**R code:** As follows.

*******************************************************************************

install.packages("rms")

library(rms)

setwd("E:\\Spine_clinical_project\\TRANSFUSION_SET\\5_Calibration")

Mydata<-read.table("input 1.0.txt",header=T,sep="\t")

ddist <- datadist(Mydata)

options(datadist="ddist")

mylog<lrm(Status~Levels_of_fusion+Total_intraoperative_EBL+Time_to_surgery+Operative_time+Preoperative_Hb,data= Mydata,x=T,y=T)

mycal<-calibrate(mylog,method="boot",B=1000)

pdf("Calibration.pdf")

plot(mycal,xlab="Nomogram-predicted probability of transfusion",ylab="Actual diagnosed transfusion (proportion)",sub=F)

dev.off()

*******************************************************************************

2.2 **ROC curve and AUC**

**Function:** We assessed the nomogram’s ability to discriminate between those who received blood transfusion and those who did not by computing the area under the receiver operating characteristic (ROC) curve (AUC). This AUC can range from 0.5 (no discriminative ability) to 1.0 (perfect discriminative ability).

**Input:** Dataset contains 885 cases parameters (Input 1.0.txt in the R code)

**Output:** The ROC curve of the prediction model is shown in Figure 5. The AUC is 0.898 (95% CI: 0.847–0.949), which suggest satisfied discrimination ability of the nomogram.

**R code:** As follows.

*******************************************************************************

library(ROCR)

library(rms)

setwd("E:\\Spine_clinical_project\\TRANSFUSION_SET\\6_ROC"

Mydata <-read.table("input.txt",header=T,sep="\t")

ddist <- datadist(Mydata)

options(datadist="ddist")

mylog<lrm(Status~Levels_of_fusion+Total_intraoperative_EBL+Time_to_surgery+Operative_time+Preoperative_Hb,data= Mydata,x=T,y=T)

mylog

pre_rate<-predict(mylog)

ROC1<- prediction(pre_rate, Mydata $Status)

ROC2<- performance(ROC1,"tpr","fpr")

AUC <- performance(ROC1,"auc")

AUC

print(AUC)

AUC<-0.898 # Here modified to calculated AUC values

pdf("ROC.pdf")

plot(ROC2,col="blue", xlab="False positive rate",ylab="True positive rate",lty=1,lwd=3,main=paste("AUC=",AUC))

abline(0,1,lty=2,lwd=3)

dev.off()

2.3 **Decision curve analysis (DCA)**

**Function:** Decision curve analysis was conducted to determine the clinical usefulness of the nomogram by quantifying the net benefits at different threshold probabilities in this dataset.

**Input:** Dataset contains 885 cases parameters (Input 1.0.txt in the R code)

**Output:** The DCA curve shows that nomogram model benefit is higher than the extreme curve (Figure 6).

**R code:** As follows.

*******************************************************************************

install.packages("rmda")

library(rms)

library(rmda)

setwd("E:\\Spine_clinical_project\\TRANSFUSION_SET\\7_DCA")

Mydata<-read.table("input.txt",header=T,sep="\t")

modul<- decision_curve(Status~ Levels_of_fusion+Total_intraoperative_EBL+Time_to_surgery+Operative_time+Preoperative_Hb,data= Mydata,

family = binomial(link ='logit'),

thresholds= seq(0,1, by = 0.01),

confidence.intervals = 0.95)

pdf("DCA.pdf")

plot_decision_curve(modul,

curve.names="Transfusion prediction nomogram",xlab="Threshold probability",

cost.benefit.axis =FALSE,col= "blue",

confidence.intervals=FALSE,

standardize = FALSE)

dev.off()

*******************************************************************************

Once again, thank you very much for your comments and suggestions. We hope the Supplementary Materials could be helpful for better understanding of the method and technical details.
